# Comparative Effects of Rehabilitation Programs After Total Knee Arthroplasty: A Modified GLA:D^®^ Program and a Lower-Limb Strengthening Program

**DOI:** 10.3390/jcm14217565

**Published:** 2025-10-25

**Authors:** Se Hee Kong, Je Yang Moon, Hyun Seung Kim, Jin Sung Bae

**Affiliations:** Department of Sports Medicine Research, Daechan Hospital, Namdong-gu, Incheon 21556, Republic of Korea; wpfid38@hanmail.net (J.Y.M.); gustmd1701@bizmeka.com (H.S.K.); jin980828@bizmeka.com (J.S.B.)

**Keywords:** GLA:D^®^, total knee arthroplasty, neuromuscular exercise, home-based exercise, lower-limb strength, rehabilitation

## Abstract

**Background:** The GLA:D^®^ program is an evidence-based intervention widely used in Western countries to improve knee function and exercise adherence among individuals with osteoarthritis. However, its application in postoperative total knee arthroplasty (TKA) populations is limited, particularly in Asia. This study evaluated two modified GLA:D^®^ interventions tailored to the Korean clinical environment. **Methods:** Patients who underwent TKA participated in one of two programs. The GLA:D-M group received therapist-supervised neuromuscular training with structured progression. The GLA:D-C group received a modified GLA:D^®^ program with additional lower-limb strengthening, primarily conducted as home-based exercise. Outcomes at 3 and 6 months included functional performance, isokinetic strength, gait speed, and psychosocial measures. Statistical significance, minimal clinically important difference (MCID), and effect sizes were used to assess clinical relevance. **Results:** Both groups improved; however, recovery patterns differed. GLA:D-M demonstrated statistically significant (*p* < 0.05) and clinically meaningful improvements in functional and psychosocial outcomes, exceeding MCID thresholds with large effect sizes. GLA:D-C showed significant gains in lower-limb strength, but many changes did not meet the MCID and did not consistently translate into higher-level functional recovery. These findings suggest that supervised neuromuscular training may facilitate more comprehensive recovery than home-based strengthening alone. **Conclusions:** Adapting the GLA:D^®^ program for TKA patients in a Korean clinical setting was feasible and beneficial. Additionally, the delivery method, particularly therapist supervision, played a vital role in maximizing outcomes. Both program content and delivery format should be considered in rehabilitation models. Larger, long-term studies are warranted to confirm these findings and explore broader clinical applications.

## 1. Introduction

Osteoarthritis (OA) is the most prevalent degenerative joint disorder and is associated with pain, swelling, and progressive structural damage in the affected joints [1]. It frequently occurs in the knee joint, resulting in a restricted range of motion, joint deformities, and severe pain. The prevalence of OA is high among the elderly, and its incidence continues to increase with the aging population [1,2]. Knee OA is a progressive disease, and when symptoms become severe, surgical treatments, such as total knee arthroplasty (TKA), are often performed.

In South Korea, the rate of TKA is 139.6 procedures per 100,000 population, which is higher than the OECD average of 110 [1,3]. In the United States, TKA is also one of the most commonly performed surgeries, with approximately 3.71 million cases reported between 2012 and 2023 [4]. The annual number of procedures currently exceeds 1.05 million [5], and it is projected to surpass 1.2 million by 2040 [6].

Chen et al. [7] reported that post-TKA rehabilitation is essential for strengthening muscles weakened by preoperative pain and inactivity, as well as for regaining control over the newly implanted joint. Scott et al. [8] highlighted that early postoperative exercises help reduce pain and restore quadriceps strength. Early exercise is also important for preventing joint contractures, reducing swelling, and restoring a normal range of motion.

Therefore, rehabilitation programs after TKA have been continuously evolving in various ways. Recently, there has been an increasing need for scientifically structured programs that address equipment and space limitations and promote patient-led participation. However, limitations remain in translating evidence-based interventions into routine clinical practice, which is a recurring issue across healthcare settings [9,10,11].

GLA:D^®^ (Good Life with osteoArthritis in Denmark) represents a standardized intervention that includes both exercise therapy and educational components, specifically designed for patients with knee and hip OA [12,13]. It aims to improve function and relieve pain through neuromuscular exercise as the core component. Neuromuscular exercises enhance balance, movement quality, muscular strength, and joint stability, thereby contributing to pain reduction.

This investigation involved modifying the structure of the GLA:D^®^ program based on its original principles [14], taking into account the specific needs of TKA patients and the Korean clinical environment. Meigh et al. [15] reported that kettlebell swing exercises may help improve knee stability in patients with OA. Although there is limited direct evidence for using kettlebells after TKA, progressive resistance training is generally considered safe when certain clinical conditions are met, such as low pain levels (VAS ≤ 3), at least 90° of knee flexion, and no joint swelling. However, high-impact movements such as deep squats, jumping, or running should be avoided during the early recovery phase [16,17,18].

Based on this, the modified program included home-based exercises to improve lower-limb strength and balance, including kettlebell swings. This study compared two rehabilitation approaches after TKA: one using the modified GLA:D^®^ program alone and the other combining it with additional home-based strength and balance exercises.

Based on this evidence, the modified program incorporated home-based lower-limb balance-strengthening exercises, including kettlebell swings.

Accordingly, the present study investigated how two distinct rehabilitation strategies influenced recovery in individuals after undergoing TKA: one involving the modified GLA:D^®^ program alone and the other incorporating additional home-based lower-limb strength and balance exercises.

## 2. Materials and Methods

### 2.1. Study Design

The study was designed as a single-center clinical trial following a randomized, prospective, and comparative approach.

**Figure 1:**
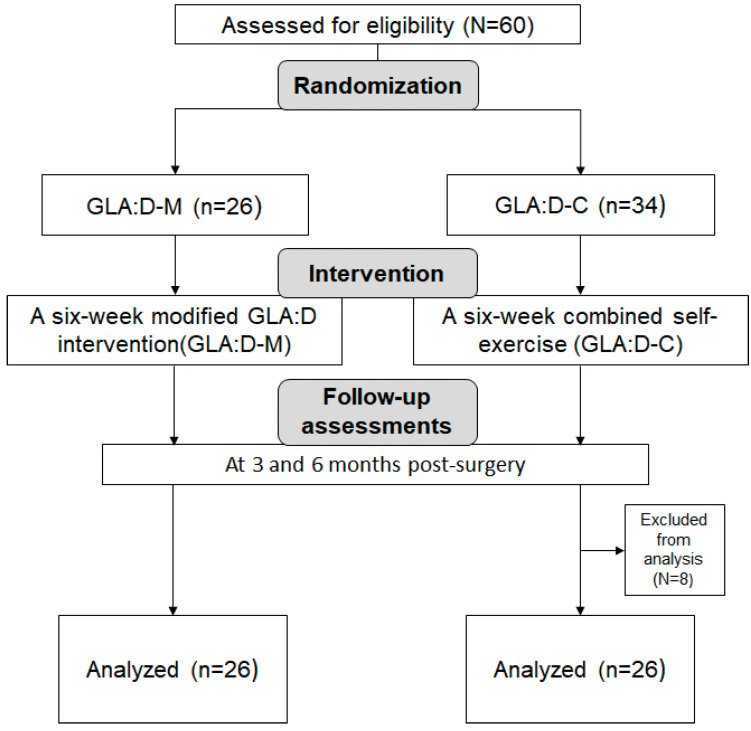
CONSORT flow chart.

Participants were randomly allocated to either the modified GLA:D intervention group (GLA:D-M) or the combined self-exercise group (GLA:D-C). The participant flow chart is shown in Figure 1.

### 2.2. Participants

Participants consisted of individuals who underwent TKA and were discharged within 2–4 weeks postoperatively. Individuals were eligible if they were at least 50 years old, had no contraindications to engaging in a rehabilitation program, and provided informed consent after receiving a full explanation of the study’s aims and procedures.

Randomization was carried out using random numbers generated with the RAND function in Microsoft Excel. According to this sequence, participants were allocated equally (1:1) into two groups: the GLA:D-M group, which followed a modified version of the GLA:D^®^ program, and the GLA:D-C group, which received the same modified program supplemented with additional lower-limb strengthening and self-directed exercises.

This study was approved by the Institutional Review Board of Eulji University (IRB No. EUIRB2024-092), and this trial was officially recorded in the Clinical Research Information Service (CRIS) maintained by the Korea Disease Control and Prevention Agency (KCT00010634).

### 2.3. Interventions

#### 2.3.1. Program Overview

The GLA:D^®^ program is an evidence-based education and neuromuscular exercise intervention developed in Denmark in 2013 for individuals with knee and hip OA [14]. The standard protocol includes two educational sessions (covering the understanding of OA, treatment options, and self-management strategies) and 12 supervised exercise sessions delivered by trained physical therapists. Each session follows a structured three-phase format: warm-up, main exercise, and cool-down. The main exercise components focus on training core stability, balance, posture, lower-limb strength, and functional movement. Its overarching goals include maintaining joint alignment, applying progressive loading, and enhancing sensorimotor control.

Neuromuscular exercises aim to improve the interactions between the muscular and nervous systems, enable stable and safe movement, reduce the risk of injury, and promote functional recovery. They also contribute to the long-term maintenance of physical function and the prevention of the recurrence of joint deterioration by improving adaptability across diverse environments. The program is based on clinical trials and systematic reviews, ensuring safety and reproducibility. Importantly, it emphasizes self-management capacity, enabling patients to continue safe and effective exercise independently after initial professional guidance [19].

In the present work, a modified version of the GLA:D^®^ program was adapted in accordance with its core principles [14], tailored to the Korean clinical environment. The original protocol, which includes standardized education and neuromuscular exercises, has been internationally adopted [14,20]. In this study, warm-up exercises using a cycle ergometer were substituted with low-impact dynamic movements (e.g., marching in place, heel raises, and lateral stepping) due to equipment limitations. Each exercise session maintained the three-phase structure of warm-up, main exercise, and cool-down.

The intervention was conducted twice a week for six weeks. Two educational sessions were provided at the beginning of the program, covering OA pathophysiology, pain management, the importance of physical activity and home exercises, and strategies for maintaining long-term adherence. All sessions were planned and led by licensed physical therapists.

The participants in the GLA:D-M group received only the modified GLA:D^®^ program. In contrast, the GLA:D-C group received the same program plus an additional 10 min of lower-limb strengthening exercises. Furthermore, GLA:D-C participants were provided with a standardized home-based exercise manual and instruction on the principles of progressive loading, and they were encouraged to perform self-directed exercises at home three to four times per week. To reflect the home-based nature of the GLA:D-C intervention, their total exercise frequency was higher than that of the GLA:D-M group. Participants in both groups were encouraged to continue exercising voluntarily after the 6-week intervention period.

A full list of exercises and detailed descriptions is provided in Supplementary Table S1.

#### 2.3.2. Intervention Fidelity and Safety Monitoring Procedures

All therapists received standardized training prior to the intervention, and their qualifications were documented. Each session followed a structured checklist to ensure adherence to the protocol. Fidelity was assessed biweekly by independent raters using a standardized checklist focused on exercise accuracy and participant engagement.

Adverse events, particularly falls, were documented by rehabilitation therapists at each session using a standardized incident report form. Participants were instructed to report any discomfort or safety concerns during the intervention.

#### 2.3.3. Statistical and Clinical Significance

To assess the clinical relevance of the observed changes, minimal clinically important difference (MCID) values from prior studies were applied. Perera et al. [21] proposed that an increase of 0.1 m/s in gait speed reflects a clinically meaningful improvement. Similarly, Wright et al. [22] identified a gain of at least two repetitions in the 30 s chair-stand test as clinically significant in older adults. Additionally, a 10% increase in isokinetic strength is commonly regarded as a threshold for meaningful enhancement in muscle performance. Effect sizes were also calculated to facilitate interpretation beyond statistical significance.

### 2.4. Pain Assessment and Schedule

#### 2.4.1. Assessment Schedule

All interventions began after a certain level of recovery following TKA, once patients were clinically eligible to participate in exercise. The effects of the interventions were assessed at 3 and 6 months postoperatively. All assessments, including questionnaires and strength measurements, were conducted by two trained evaluators using standardized methods.

#### 2.4.2. Pain Assessment

##### Visual Analog Scale (VAS)

VAS, originally introduced by Huskisson, is a widely used tool in clinical settings for pain evaluation [23]. It comprises a 10 cm visual scale, ranging from “no pain” (10) to “worst possible pain.” The participants recorded the spot on the scale that most closely represented their pain level intensity. Higher scores indicate more severe pain [24].

##### Keele STarT MSK Tool (STarT MSK)

The STarT MSK tool, developed by Campbell et al., is a screening tool used to predict pain and disability in patients with musculoskeletal disorders [25]. It includes ten items that assess physical and psychological risk factors, yielding a score between 0 and 12 [26]. Scores of 0–4 indicate low risk, 5–8 indicate moderate risk, and 9–12 indicate high risk.

#### 2.4.3. Knee Function Assessment

##### Knee Injury and Osteoarthritis Outcome Score-12 (KOOS-12)

Developed by Gandek et al., the KOOS-12 is a short-form tool derived from the original KOOS-42 and designed to assess knee function [27]. The assessment covers pain, function, and quality of life, with 12 items rated on a 5-point Likert scale from 0 (no problem) to 4 (most severe problem). Raw scores are transformed into a 0–100 scale, with higher scores indicating better knee function [28].

#### 2.4.4. Psychological Distress Assessment

##### Kessler Psychological Distress Scale (K10)

The K10, developed by Kessler et al., is a 10-item questionnaire used to assess psychological distress symptoms occurring in the last 4 weeks [29]. Each item is scored on a 5-point scale, producing total scores between 10 and 50, with higher values indicating more severe psychological distress [30].

#### 2.4.5. Health-Related Quality of Life Assessment

##### EQ-5D

Developed by the EuroQol Group, EQ-5D evaluates health-related quality of life [31]. It includes five dimensions: mobility, self-care, usual activities, pain/discomfort, and anxiety/depression, which are assessed using a Likert scale. Higher scores indicate a better quality of life [32].

#### 2.4.6. Muscle Strength Assessment

##### Quadriceps Strength

Isokinetic strength of the quadriceps was measured using a Biodex System 4 (Biodex Medical Systems, Inc., Long Island, NY, USA). Testing was performed at angular velocities of 60°/s and 180°/s. The participants were seated with their hips and knees secured. The peak torque at each velocity was recorded in Newton meters (Nm). The reliability of this equipment has been reported as good to excellent in previous studies [33].

#### 2.4.7. Physical Performance Tests

##### 4 × 10 m Fast Paced Walk Test (40 m FPWT)

The 40 m walk test, proposed by Bohannon et al., assesses walking ability [34]. Following the OARSI recommendations [35], participants walked a 10 m distance back and forth four times (a total of 40 m) as fast and safely as possible. The time was recorded and converted to walking speed (m/s).

##### 30 s Chair-Stand Test (30 s CST)

Lower-limb strength and dynamic balance were assessed using the 30 s CST, developed by Jones and Rikli [36]. The participants performed repeated sit-to-stand motions from an armless chair (approx. 43 cm in height) for 30 s. The number of completed repetitions was recorded [37].

### 2.5. Sample Size Calculation

Using G*Power 3.1 for a two-tailed independent t-test with α = 0.05, power (1–β) = 0.90, and effect size = 0.80, the minimum required sample size was calculated to be 21 participants per group. To account for potential dropouts, 60 participants were initially recruited. After excluding dropouts, 52 participants (26 per group) were included in the final analysis.

Although the sample size calculation was based on parametric assumptions, the final data did not meet the normality criteria. Therefore, non-parametric tests (Mann–Whitney U and Wilcoxon signed-rank tests) were used to analyze between-group and within-group differences, respectively.

### 2.6. Statistical Analysis

Analyses were conducted using SPSS (version 22.0; IBM Corp., Armonk, NY, USA). Differences between the two groups were assessed with the Mann–Whitney U test. Within-group changes were analyzed using the Wilcoxon signed-rank test. Statistical significance was set at *p* < 0.05.

## 3. Results

Participant characteristics at the 3-month assessment are presented in Table 1. Since no true pre-intervention baseline data were collected, the 3-month assessment served as the reference point. In the GLA:D-M group, 26.9% were male and 73.1% were female, while in the GLA:D-C group, 30.8% were male and 69.2% were female. The sex distribution between the groups was comparable, with no significant difference detected (*p* > 0.05). The mean age was 66.62 ± 7.79 years in the GLA:D-M group and 68.59 ± 5.59 years in the GLA:D-C group (*p* > 0.05). The mean body mass index (BMI) was 24.95 ± 1.02 in the GLA:D-M group and 25.35 ± 1.02 in the GLA:D-C group, with no significant difference (*p* > 0.05). These results confirmed the demographic and anthropometric equivalence of the two groups.

Pre-intervention baseline data were not collected; therefore, the 3-month assessment served as the reference point for group comparison. At this time point, KOOS-12 (*p* < 0.001) and STarT MSK (*p* < 0.05) differed significantly between groups, indicating that these variables were not comparable. The comparison of changes between the two groups from 3 to 6 months is reported in Table 2. Because only KOOS-12 and STarT MSK showed significant group differences at the 3-month assessment, ANCOVA was conducted for these two outcomes using the 3-month scores as covariates. The results are presented in Table 3.

Between-group differences in change scores from 3 to 6 months were first examined using the Mann–Whitney U test. Significant group differences were observed in KOOS-12, STarT MSK, and the 30 s chair-stand test (30 s CST), with the direction favoring GLA:D-M, while knee extensor strength at 180°/s favored GLA:D-C. No significant between-group differences were found for VAS, EQ-5D, K10, FPWT, or strength at 60°/s, indicating that most outcomes showed minimal group effects.

Since KOOS-12 and STarT MSK differed between groups at the 3-month assessment, ANCOVA was conducted to adjust for these baseline values. After controlling for the 3-month scores, the adjusted KOOS-12 remained higher in GLA:D-M (F(1,49) = 4.231, *p* = 0.045, partial η^2^ = 0.079), while the adjusted STarT MSK remained lower (F(1,49) = 8.884, *p* = 0.005, partial η^2^ = 0.151), confirming small-to-moderate between-group differences in favor of GLA:D-M. However, the magnitude of these effects was limited, and no single program demonstrated superiority across all outcomes.

The within-group changes from 3 to 6 months in the GLA:D-M group are summarized in Table 4.

Between the third and sixth months, participants in the GLA:D-M group demonstrated statistically significant improvements across multiple domains. Notable progress was observed in pain levels (*p* < 0.001, r = 0.28), musculoskeletal risk (*p* < 0.001, r = 0.42), functional ability (*p* < 0.001, r = 0.45), and psychological distress (*p* = 0.001, r = 0.33). Although changes in quality of life did not reach statistical significance (*p* = 0.097), the moderate effect size (r = 0.31) indicates a positive trend.

Conversely, the GLA:D-C group showed no statistically significant changes in any of the assessed outcomes, as all *p*-values were greater than 0.05. Effect sizes in this group ranged from negligible to small (r = 0.02–0.20), indicating minimal impact.

These findings suggest that the modified GLA:D program alone was effective in producing consistent and clinically meaningful improvements over time. In contrast, the addition of home-based strengthening exercises did not lead to observable within-group changes during the 3–6-month period.

The within-group changes from 3 to 6 months in the GLA:D-C group are summarized in Table 5. In the GLA:D-M group, 40 m FPWT significantly improved (*p* < 0.001, r = 0.72) and exceeded the MCID (+0.10 m/s), while the GLA:D-C group also showed significant improvement (*p* = 0.001, r = 0.69, MCID achieved), although the magnitude was slightly smaller.

Only the GLA:D-M group demonstrated a significant increase in 30 s CST (*p* < 0.001, r = 0.78, MCID achieved), whereas the GLA:D-C group showed no significant change (*p* = 0.399).

At 60°/s, both extensor and flexor strength increased significantly in both groups (all *p* ≤ 0.003, r = 0.63–0.74), and both met MCID criteria.

At 180°/s, the GLA:D-M group showed significant and clinically meaningful gains in both extensor and flexor strength (all *p* < 0.001, r = 0.66–0.68, MCID achieved), while the GLA:D-C group demonstrated smaller improvements, with MCID not consistently achieved (extensor: *p* = 0.004; flexor: *p* = 0.036).

**Figure 2:**
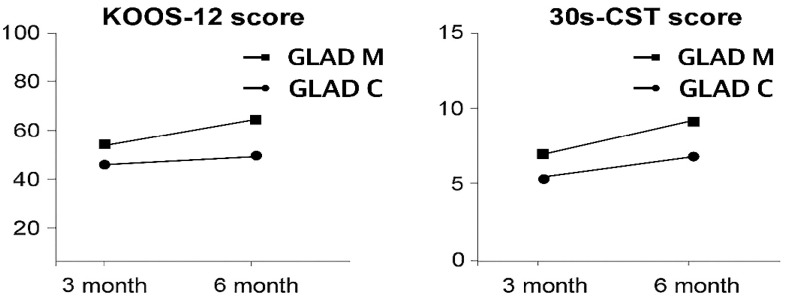
Changes in KOOS-12 and 30 s CST scores.

Overall, the GLA:D-M group demonstrated more consistent, larger, and clinically meaningful improvements across most outcomes, whereas the GLA:D-C group showed strength gains but limited functional improvements. The changes in KOOS-12 and 30 s CST scores are presented in Figure 2. 

## 4. Discussion

The GLA:D^®^ exercise program was originally developed in Denmark and has since been adopted in various countries. Designed with scientific rigor, it aims to improve knee joint function in older adults and support participants in consistently performing home-based exercises. However, since the program has not been fully implemented in Korea, this study applied a modified GLA:D^®^ program tailored to the domestic clinical environment to evaluate its effectiveness.

The analysis revealed improvements in both the GLA:D-M and GLA:D-C groups, although the recovery patterns differed. The GLA:D-M group showed significant and broader enhancements in overall functional performance and psychosocial outcomes, whereas the GLA:D-C group demonstrated more pronounced gains in lower-limb strength. This indicates that the structure and delivery method of each program influenced the outcomes. The GLA:D-M program was conducted by physical therapists who utilized supervised neuromuscular exercises with a structured progression, likely to ensure accurate movement execution and consistent exercise quality. In contrast, the GLA:D-C program incorporated a modified GLA:D^®^ approach with additional lower-limb strengthening; however, most of the strengthening exercises were self-directed home activities. Consequently, while lower-limb strength improved, these gains did not fully translate into overall functional recovery.

In addition, GLA:D-M demonstrated statistically significant improvements in functional and psychosocial outcomes (*p* < 0.05), and these changes exceeded MCID thresholds and showed large effect sizes, confirming both statistical and clinical relevance. This pattern is consistent with Skou et al. [14], who reported improvements in pain, function, and quality of life following the GLA:D^®^ program. In contrast, GLA:D-C showed significant gains mainly in isokinetic strength, and several of these changes did not reach MCID or lead to meaningful functional improvements. This suggests that lower-limb strengthening alone may be insufficient without supervised integration into functional tasks.

Pihl et al. [38] also demonstrated improvements in gait speed, pain, and quality of life after the GLA:D^®^ program. The GLA:D-M group in our study showed similar functional trends, but quality of life did not exhibit a statistically significant change. This may be due to the short follow-up period and potential ceiling effects in early postoperative TKA patients. Pihl et al. [38] primarily reported quality of life improvement after 12 months, indicating that longer follow-up may be necessary to detect changes in this population.

Although most GLA:D^®^ research has been conducted in Western countries, several Asian studies have investigated neuromuscular-based interventions in TKA rehabilitation. In Korea, Yoon et al. [39] found that neuromuscular electrical stimulation improved quadriceps strength and accelerated functional recovery. In Japan, Sakaki et al. [40] reported that proprioceptive neuromuscular facilitation enhanced strength, proprioception, and stability. These studies support the value of neuromuscular approaches. However, these interventions focused on specific muscle groups or single techniques. In contrast, the present study used a comprehensive neuromuscular framework based on GLA:D^®^ principles, targeting movement quality, balance, coordination, and functional task performance. This broader approach may explain why GLA:D-M produced more consistent and meaningful recovery than localized strengthening alone.

From a clinical perspective, neuromuscular training helped patients use their strength more effectively in daily movements. Therapist supervision and structured progression likely improved adherence and movement precision. GLA:D-C enhanced muscle torque through additional strengthening, but the self-directed nature of the exercises may have limited the transfer of gains to functional performance. This highlights the importance of how exercises are delivered, not just which exercises are performed.

Finally, the modified GLA:D^®^ program used in this study aligns with international efforts to adapt evidence-based rehabilitation models to different healthcare systems. In Switzerland, the program was successfully implemented by adjusting delivery methods to address workforce and institutional barriers [41]. Furthermore, both face-to-face and telehealth formats have shown comparable outcomes and high patient satisfaction [42], suggesting that preserving the core neuromuscular principles while allowing flexibility in delivery is effective. By applying a tailored GLA:D^®^ model in an Asian TKA population, this study adds meaningful evidence for global adaptation and clinical feasibility.

Overall, this study demonstrates that a neuromuscular-based, therapist-supervised GLA:D^®^ program can yield statistically significant and clinically meaningful improvements after TKA. GLA:D-M led to a more comprehensive recovery than GLA:D-C, indicating that integrated neuromuscular training may be more effective than strength-focused, self-directed additions alone. These findings have practical implications for postoperative rehabilitation and support further research on long-term outcomes and optimized delivery strategies.

Despite the strengths of this study, several limitations should be acknowledged. First, although group comparability was confirmed at the 3-month postoperative point, the lack of preoperative baseline data restricts insights into early recovery dynamics. Second, while participants in the home-based exercise group were advised to continue training independently, their actual engagement was not systematically monitored. Incorporating digital tracking or self-report tools in future research may help address this gap.

Third, although outcome assessments were conducted by blinded evaluators, the lack of allocation concealment during group assignment may have introduced bias. Additionally, the relatively small sample size and single-center design limit the external validity of the findings. To enhance generalizability and assess long-term outcomes, future studies should consider larger, multicenter trials with extended follow-up periods. Evaluating the feasibility of implementing the modified GLA:D^®^ program in broader community settings would also be beneficial.

## 5. Conclusions

This study explored the potential effectiveness of a modified GLA:D^®^ program tailored to the Korean clinical setting to aid postoperative rehabilitation after TKA. The structured intervention, which emphasizes neuromuscular exercise, appears to support improvements in overall functional recovery. In contrast, the approach that combines home-based exercise with lower-limb strengthening showed notable gains in muscle strength. The findings suggest that the GLA:D-M program may enhance functional performance more broadly, while the GLA:D-C program may be better suited for improving muscular strength. These results indicate that GLA:D^®^-based programs could be adapted to align with specific rehabilitation goals, providing preliminary insights for the development of diverse and goal-oriented rehabilitation strategies.

## Figures and Tables

**Table 1:** Group characteristics at 3 months.

Variable	GLA:D-M (n = 26)	GLA:D-C (n = 26)	Mann–Whitney U	*p*-Value
Sex, n (%)				
Male	7 (26.9%)	8 (30.8%)		0.760
Female	19 (73.1%)	18 (69.2%)		
Age				
Mean ± SD	66.62 ± 7.79	68.59 ± 5.59		0.263
Median [min–max]	66.5 [51–81]	69.0 [57–79]		
BMI				
Mean ± SD	24.95 ± 1.02	25.35 ± 1.02		0.592
Median [min–max]	24.95 [23.1–26.8]	25.35 [23.6–27.7]		0.592
VAS			250.000	0.100
STarT MSK			216.500	0.025 *
KOOS-12			8.500	0.000 ***
EQ-5D			251.500	0.111
K10			283.500	0.315
40 m FPWT			296.000	0.442
30 s CST			268.000	0.197
60 s Extensor			299.000	0.475
60 s Flexor			292.500	0.405
180 s Extensor			330.500	0.891
180 s Flexor			325.500	0.819

Values are presented as numbers (%), mean ± standard deviations, or medians [minimum–maximum]. BMI, body mass index, kg/m^2^. The chi-square test was used for categorical variables, while the Mann–Whitney U test was employed for continuous variables. Values for the 3-month assessment test are presented as Mann–Whitney U statistics and *p*-values. *p* < 0.05 *, *p* < 0.001 *** VAS = visual analog scale; sTarT = Keele STarT Musculoskeletal Tool; KOOS-12 = Knee Injury and Osteoarthritis Outcome Score-12; EQ-5D = EuroQol 5-Dimension; K10 = Kessler Psychological Distress Scale-10; 40 m Walk = 40 m fast-paced walk test; 30 s Chair Stand = 30 s chair-stand test; Ext = extensor; Flex = flexor.

**Table 2:** Comparison of change scores between groups from 3 to 6 months (Mann–Whitney U test).

Variable	Mann–Whitney U	Wilcoxon W	Z	*p*-Value
VAS	245.000	596.000	−1.726	0.084
STarT MSK	161.000	512.000	−3.273	0.001 **
KOOS-12	19.000	370.000	−5.839	0.000 ***
EQ-5D	293.000	644.000	−0.827	0.408
K10	279.500	630.500	−1.076	0.282
40 m FPWT	296.500	647.500	−0.760	0.447
30 s CST	153.500	504.500	−3.387	0.001 **
60 s Extensor	266.000	617.000	−1.318	0.188
60 s Flexor	333.500	684.500	−0.082	0.934
180 s Extensor	213.000	564.000	−2.288	0.022 *
180 s Flexor	246.000	597.000	−1.684	0.092

*p* < 0.05 *, *p* < 0.01 **, *p* < 0.001 *** (Mann–Whitney U test). VAS = Visual Analog Scale; STarT MSK = Keele STarT Musculoskeletal Tool; KOOS-12 = Knee Injury and Osteoarthritis Outcome Score-12; EQ-5D = EuroQol 5-Dimension; K10 = Kessler Psychological Distress Scale-10; 40 m FPWT = 40 m fast-paced walk test; 30 s CST = 30 s chair-stand test; Extensor = Quadriceps Extensor Strength; Flexor = Hamstring Flexor Strength.

**Table 3:** ANCOVA results adjusted for 3-month assessment.

Outcome	Adjusted Mean (Intervention)	Adjusted Mean (Control)	F	*p*-Value	Partial η^2^
KOOS-12	66.19	64.70	4.231	0.045 *	0.079
STarT MSK	3.50	4.07	8.884	0.005 **	0.151

*p* < 0.05 *, *p* < 0.01 **, adjusted means, F, *p*-values, and effect sizes from ANCOVA controlling for the 3-month assessment. KOOS-12 = Knee Injury and Osteoarthritis Outcome Score-12; STarT MSK = Keele STarT Musculoskeletal Tool.

**Table 4:** Changes in clinical outcomes (Wilcoxon signed-rank test).

Variable	Group	n	3M Mean ± SD	6M Mean ± SD	ΔMean ± SD	*p*-Value	Effect Size (r)
VAS	GLA:D-M	26	3.46 ± 1.77	2.85 ± 1.76	–0.61 ± 1.45	0.00 **	0.28
	GLA:D-C	26	3.62 ± 1.86	3.42 ± 1.73	–0.19 ± 1.30	1.000	0.09
STarT MSK	GLA:D-M	26	7.88 ± 2.83	6.19 ± 2.64	–1.69 ± 2.16	0.000 ***	0.42
	GLA:D-C	26	9.50 ± 2.70	8.96 ± 2.56	–0.54 ± 1.70	0.380	0.17
KOOS-12	GLA:D-M	26	54.85 ± 11.61	63.58 ± 12.68	+8.73 ± 9.54	0.000 ***	0.45
	GLA:D-C	26	47.42 ± 10.61	50.19 ± 11.57	+2.77 ± 6.87	0.855	0.22
EQ-5D	GLA:D-M	26	0.71 ± 0.10	0.76 ± 0.09	+0.05 ± 0.09	0.097	0.31
	GLA:D-C	26	0.69 ± 0.11	0.72 ± 0.10	+0.03 ± 0.08	0.270	0.18
K10	GLA:D-M	26	18.38 ± 5.22	16.62 ± 5.06	–1.77 ± 3.64	0.001 **	0.33
	GLA:D-C	26	19.38 ± 5.15	18.54 ± 4.92	–0.85 ± 2.69	0.130	0.20

Wilcoxon signed-rank test: *p* < 0.01 **, *p* < 0.001 *** VAS = Visual Analog Scale; STarT MSK = Keele STarT Musculoskeletal Tool; KOOS-12 = Knee Injury and Osteoarthritis Outcome Score-12; EQ-5D = EuroQol 5-Dimension; K10 = Kessler Psychological Distress Scale-10.

**Table 5:** Functional outcomes and MCID achievement (Wilcoxon signed-rank test).

Variable		Group	n	3M Mean ± SD	6M Mean ± SD	ΔMean ± SD	*p*-Value	Effect Size r	MCID-Based	MCID Achieved
40 m FPWT		GLA:D-M	26	1.16 ± 0.18	1.37 ± 0.17	+0.21 ± 0.11	0.000 ***	0.72	+0.10 m/s	Y
		GLA:D-C	26	1.17 ± 0.11	1.35 ± 0.19	+0.18 ± 0.10	0.001 **	0.69	+0.10 m/s	Y
30 s CST		GLA:D-M	26	11.35 ± 2.68	15.62 ± 2.99	+4.27 ± 2.6	0.000 ***	0.78	+2 reps	Y
		GLA:D-C	26	11.00 ± 2.54	11.54 ± 2.77	+0.54 ± 1.21	0.399	0.30	+2 reps	N
60°/ s	Ext	GLA:D-M	26	53.77 ± 19.42	70.77 ± 21.29	+17.00 ± 10.52	0.000 ***	0.74	≥10%	Y
		GLA:D-C	26	56.31 ± 18.88	66.13 ± 21.51	+9.82 ± 9.02	0.003 **	0.63	≥10%	Y
	Flex	GLA:D-M	26	33.50 ± 11.82	43.66 ± 12.90	+10.16 ± 7.85	0.003 **	0.71	≥10%	Y
		GLA:D-C	26	35.12 ± 12.44	45.35 ± 13.50	+10.23 ± 8.02	0.001 **	0.69	≥10%	Y
180°/s	Ext	GLA:D-M	26	40.42 ± 14.10	51.50 ± 15.22	+11.07 ± 8.12	0.000 ***	0.68	≥10%	Y
		GLA:D-C	26	39.69 ± 13.88	44.48 ± 14.25	+4.79 ± 6.32	0.004 **	0.54	≥10%	Y
	Flex	GLA:D-M	26	29.63 ± 10.75	38.46 ± 12.07	+8.83 ± 7.21	0.001 **	0.66	≥10%	Y
		GLA:D-C	26	31.23 ± 11.02	35.00 ± 11.84	+3.77 ± 5.66	0.036 *	0.46	≥10%	Y

Wilcoxon signed-rank test, *p* < 0.05 *, *p* < 0.01 **, *p* < 0.001 *** FPWT = 40 m fast-paced walk test; 30 s CST = 30 s chair-stand test; Ext = Extensor; Flex = Flexor. MCID Achieved indicates whether the change in outcome meets or exceeds the minimal clinically important difference (MCID).

## Data Availability

The data supporting the findings of this study are openly accessible via Figshare at the following https://doi.org/10.6084/m9.figshare.30228736.

## Supplementary Materials

The following supporting information can be downloaded at https://www.mdpi.com/article/10.3390/jcm14217565/s1. Table S1-1: Weekly progression of the modified GLA:D^®^ exercise program (GLA:D-M); Table S1-2: Weekly progression of the modified GLA:D^®^ exercise program (GLA:D-C).

## References

[1] Tong L., Yu H., Huang X., Shen J., Xiao G., Chen L., Wang H., Xing L., Chen D. (2022). Current understanding of osteoarthritis pathogenesis and relevant new approaches. Bone Res..

[2] Choi H.Y. (2020). Factors influencing health-related quality of life among Korean seniors with osteoarthritis: Focusing on 10-year duration with osteoarthritis disease. Korean J. Adult Nurs..

[3] Organization for Economic Cooperation and Development (2022). OECD Health Statistics 2022.

[4] American Joint Replacement Registry (AJRR) (2024). 11th Annual Report 2024: Hip and Knee Arthroplasty Data.

[5] Heckmann N.D., Mayfield C.K., Richardson M.K., Liu K.C., Wang J.C., Piple A.S., Stambough J.B., Oakes D.A., Christ A.B., Lieberman J.R. (2024). An updated estimate of total hip and total knee arthroplasty utilization in the United States. Arthroplast. Today.

[6] Shichman I., Roof M., Askew N., Nherera L., Rozell J.C., Seyler T.M., Schwarzkopf R. (2023). Projections and epidemiology of primary hip and knee arthroplasty in Medicare patients to 2040–2060. JBJS Open Access.

[7] Chen S.R., Chen C.S., Lin P.C. (2014). The effect of educational intervention on the pain and rehabilitation performance of patients who undergo a total knee replacement. J. Clin. Nurs..

[8] Scott D.F. (2011). Knee Joint Replacement Surgery Post-Operative Exercise Program. https://www.orthospecialtyclinic.com/pdf/osc-knee-repl-exercise-pro.pdf.

[9] Braithwaite J., Wears R.L., Hollnagel E. (2018). When complexity science meets implementation science: A theoretical and empirical analysis of systems change. BMC Med..

[10] Dixon-Woods M. (2012). Ten challenges in improving quality in healthcare: Lessons from the Health Foundation’s programme evaluations and relevant literature. BMJ Qual. Saf..

[11] Fischer F., Lange K., Klose K., Greiner W., Kraemer A. (2016). Barriers and strategies in guideline implementation—A scoping review. Healthcare.

[12] Kongsted A., Hartvigsen J., Boyle E., Ris I., Kjaer P., Thomassen L., Vach W. (2019). GLA: D^®^ Back: Group-based patient education integrated with exercises to support self-management of persistent back pain—Feasibility of implementing standardised care by a course for clinicians. Pilot. Feasibility Stud..

[13] Kongsted A., Hartvigsen J., Boyle E., Ris I., Kjaer P., Thomassen L., Vach W. Group-based patient education and exercises to support self-management of persistent/recurrent back pain. Feasibility of an implementation-effectiveness trial. Proceedings of the World Confederation for Physical Therapy Congress (WCPT 2019).

[14] Skou S.T., Roos E.M. (2017). Good Life with osteoArthritis in Denmark (GLA: D™): Evidence-based education and supervised neuromuscular exercise delivered by certified physiotherapists nationwide. BMC Musculoskelet. Disord..

[15] Meigh N.J., Keogh J.W.L., Schram B., Hing W.A. (2019). Kettlebell training in clinical practice: A scoping review. BMC Sports Sci. Med. Rehabil..

[16] American Academy of Orthopaedic Surgeons Total Knee Replacement Exercise Guide. OrthoInfo. https://orthoinfo.aaos.org/en/recovery/total-knee-replacement-exercise-guide/.

[17] Sports Surgery Clinic (2021). Total Knee Replacement Rehabilitation Protocol; PDF. https://www.sportssurgeryclinic.com/wp-content/uploads/2021/09/TKR-Protocol-Final.pdf.

[18] Royal Berkshire NHS Foundation Trust (2024). Advice and Exercises After Total Knee Replacement; PDF. https://www.royalberkshire.nhs.uk/media/ww5bknsi/advice-and-exercises-after-total-knee-replacement_nov24.pdf.

[19] Roos E.M., Barton C.J., Davis A.M., McGlasson R., Kemp J.L., Crossley K.M., Liu Q., Lin J., Skou S.T. (2018). GLA:D^®^ to have a high-value option for patients with knee and hip arthritisacross four continents: Good Life with osteoArthritis from Denmark. Br. J. Sports Med..

[20] Roos E.M., Grønne D.T., Skou S.T., Zywiel M.G., McGlasson R., Barton C.J., Kemp J.L., Crossley K.M., Davis A.M. (2021). Immediate outcomes following the GLA: D^®^ program in Denmark, Canada and Australia. A longitudinal analysis including 28,370 patients with symptomatic knee or hip osteoarthritis. Osteoarthr. Cartil..

[21] Perera S., Mody S.H., Woodman R.C., Studenski S.A. (2006). Meaningful Change and Responsiveness in Common Physical Performance Measures in Older Adults. J. Am. Geriatr. Soc..

[22] Wright A.A., Cook C.E., Baxter G.D., Dockerty J.D., Abbott J.H. (2011). A Comparison of 3 Methodological Approaches to Defining Major Clinically Important Improvement of 4 Performance Measures in Patients with Hip Osteoarthritis. J. Orthop. Sports Phys. Ther..

[23] Huskisson E.C. (1974). Measurement of pain. Lancet.

[24] Alghadir A.H., Anwer S., Iqbal A., Iqbal Z.A. (2018). Test–retest reliability, validity, and minimum detectable change of visual analog, numerical rating, and verbal rating scales for measurement of osteoarthritic knee pain. J. Pain. Res..

[25] Campbell P., Hill J.C., Protheroe J., Afolabi E.K., Lewis M., Beardmore R., Hay E.M., Mallen C.D., Bartlam B., Saunders B. (2016). Keele Aches and Pains Study protocol: Validity, acceptability, and feasibility of the Keele STarT MSK tool for subgrouping musculoskeletal patients in primary care. J. Pain. Res..

[26] Carvajal-Parodi C., Ulloa-Díaz D., Romero-Vera L., Andrades-Ramírez O., Guede-Rojas F., Ponce-González J.G. (2025). Immersive Virtual Reality-Based Exercise for Pain Management in Fibromyalgia: An Exploratory Study with Risk of Poor Outcomes Stratification. Appl. Sci..

[27] Gandek B., Roos E.M., Franklin P.D., Ware J.E. (2019). Item selection for 12-item short forms of the Knee injury and Osteoarthritis Outcome Score (KOOS-12) and Hip disability and Osteoarthritis Outcome Score (HOOS-12). Osteoarthr. Cartil..

[28] Stokholm R., Larsen P., Petruskevicius J., Rölfing J.D., Rasmussen M.K., Jensen S.S., Elsoe R. (2025). Knee Injury and Osteoarthritis Outcome Score for Tibial Shaft Fractures: Validity, Reliability, Responsiveness, and Minimal Clinically Important Difference. Orthopedics.

[29] Kessler R.C., Barker P.R., Colpe L.J., Epstein J.F., Gfroerer J.C., Hiripi E., Howes M.J., Normand S.L.T., Manderscheid R.W., Walters E.E. (2003). Screening for serious mental illness in the general population. Arch. Gen. Psychiatry.

[30] Zhou Z., Yuan J., Zhang Y., Wu P., Lv W. (2025). Evaluation of psychological status in early-stage breast cancer outpatients: A cross-sectional study utilizing the Kessler 10 Scale. BMC Psychiatry.

[31] The EuroQol Group (1990). EuroQol-a new facility for the measurement of health-related quality of life. Health Policy.

[32] Haridoss M., Bagepally B.S., Natarajan M. (2021). Health-related quality of life in rheumatoid arthritis: Systematic review and meta-analysis of EuroQoL (EQ-5D) utility scores from Asia. Int. J. Rheum. Dis..

[33] Tuominen J., Leppänen M., Jarske H., Pasanen K., Vasankari T., Parkkari J. (2023). Test–retest reliability of isokinetic ankle, knee and hip strength in physically active adults using Biodex System 4 Pro. Methods Protoc..

[34] Bohannon R.W. (1997). Comfortable and maximum walking speed of adults aged 20–79 years: Reference values and determinants. Age Ageing.

[35] Dobson F., Hinman R.S., Roos E.M., Abbott J.H., Stratford P., Davis A.M., Buchbinder R., Snyder-Mackler L., Henrotin Y., Thumboo J. (2013). OARSI recommended performance-based tests to assess physical function in people diagnosed with hip or knee osteoarthritis. Osteoarthr. Cartil..

[36] Jones C.J., Rikli R.E., Beam W.C. (1999). A 30-s chair-stand test as a measure of lower body strength in community-residing older adults. Res. Q. Exerc. Sport..

[37] Ramalho R.B., Chaves T.C., Terluin B., Selistre L.F.A. (2025). Minimal important changes of common outcome measures of physical function in individuals with knee osteoarthritis: A prospective clinical study. Arch. Phys. Med. Rehabil..

[38] Pihl K., Roos E.M., Taylor R.S., Grønne D.T. (2021). Associations between comorbidities and immediate and one-year outcomes following supervised exercise therapy and patient education—A cohort study of 24,513 individuals with knee or hip osteoarthritis. Osteoarthr. Cartil..

[39] Yoon J.H., Jo S., Kim S.-H. (2017). Neuromuscular electrical stimulation therapy after knee surgery: A systematic review. J. Korean Med. Assoc..

[40] Sakai S., Watanabe M., Itoh Y., Sato N., Hamaguchi N., Fukuta M. (2025). Effects of Neuromuscular Electrical Stimulation on Quadriceps Muscle Strength in the Early Postoperative Period After Total Knee Arthroplasty. Phys. Ther. Res..

[41] Hinteregger A., Niedermann K., Wirz M. (2023). The feasibility, facilitators, and barriers in the initial implementation phase of ‘good life with osteoarthritis in Denmark’ (GLA:D^®^) in Switzerland: A cross-sectional survey. BMC Health Serv. Res..

[42] Ezzat A.M., Kemp J.L., Heerey J.J., Pazzinatto M.F., De Oliveira Silva D., Dundules K., Francis M., Barton C.J. (2025). Implementation of the Good Life with osteoArthritis in Denmark (GLA: D^®^) program via telehealth in Australia: A mixed-methods program evaluation. J. Telemed. Telecare.

