# Supplementary material for: Comparative Effects of Rehabilitation Programs After Total Knee Arthroplasty: A Modified GLA:D® Program and a Lower-Limb Strengthening Program
Source: J Clin Med. 2025 Oct 25;14(21):7565. doi: 10.3390/jcm14217565 (PMC12610574; doi:10.3390/jcm14217565)
Supplement: Supplementary file 1 [file jcm-14-07565-s001.zip › jcm-3920102-supplementary.pdf]

Table S1-1. Weekly progression of the modified GLA:D® exercise program (GLA:D-M)

Week	Category	Exercise	Repetitions/Sets
Warm-up	–	Low marching in place or side step	1–2 min
1	Core	Pelvic bridge	15 s × 3
	Balance	Side step (band)	15 reps × 2
	Posture	Weight shift	10 reps × 2
2	Strength	Sit-to-stand	8 reps × 2
	Core	Plank (intro)	10 s × 3
	Hip	Band abduction	15 reps × 2
3	Strength	Step-up	8 reps × 3
	Core	Pelvic bridge	15 s × 3
	Control	Controlled lunge	8 reps × 2
4	Core	Plank hold	20 s × 3
	Strength	Step lunge	10 reps × 3
	Posture	Alignment drill	1 min × 2
5	Strength	Progressive lunge	12 reps × 3
	Power	Fast sit-to-stand	15 reps × 2
	Balance	Balance hold	30 s
6	Functional circuit	Bridge, sit-to-stand, balance	3 sets
Cooling down	–	Stretching (major lower-limb muscles)	5 min

Table S1-2. Weekly progression of the modified GLA:D® exercise program (GLA:D-C)

Week	Category	Exercise	Repetitions/Sets
Warm-up	–	Low marching in place or side step	1–2 min
1	Strength	Goblet squat (body weight)	8 reps × 2
	Core	Bridge	15 s × 3
	Balance	Side step	15 reps × 2
2	Core	Plank	10 s × 3
	Strength	Deadlift (intro)	10 reps × 2
	Strength	Sit-to-stand	10 reps × 2
3	Strength	Goblet squat (light kettlebell)	10 reps × 3
	Power	Swing (intro)	8 reps × 2
	Balance	Balance drill	30 s
4	Strength	Step lunge with rotation	10 reps × 3
	Balance	Single-leg deadlift	10 reps × 2
	Core	Plank	20 s × 2
5	Power	Swing + squat combo	12 reps × 3
	Strength	Dynamic step-up	12 reps × 3
6	Functional circuit	Swing, lunge, squat	3 sets
Cooling down	–	Stretching (major lower-limb muscles)	5 min
